# Serralysin family metalloproteases protects *Serratia marcescens* from predation by the predatory bacteria *Micavibrio aeruginosavorus*

**DOI:** 10.1038/s41598-018-32330-4

**Published:** 2018-09-19

**Authors:** Carlos J. Garcia, Androulla Pericleous, Mennat Elsayed, Michael Tran, Shilpi Gupta, Jake D. Callaghan, Nicholas A. Stella, Jonathan M. Franks, Patrick H. Thibodeau, Robert M. Q. Shanks, Daniel E. Kadouri

**Affiliations:** 1 Department of Oral Biology, Rutgers School of Dental Medicine, Newark, NJ 07103 USA; 2 Department of Ophthalmology, Charles T. Campbell Laboratory of Ophthalmic Microbiology, University of Pittsburgh, Pittsburgh, PA 15213 USA; 3 Center for Biologic Imaging, University of Pittsburgh, Pittsburgh, PA 15213 USA; 4 Department of Microbiology and Molecular Genetics, University of Pittsburgh, Pittsburgh, PA 15221 USA

## Abstract

*Micavibrio aeruginosavorus* is an obligate Gram-negative predatory bacterial species that feeds on other Gram-negative bacteria by attaching to the surface of its prey and feeding on the prey’s cellular contents. In this study, *Serratia marcescens* with defined mutations in genes for extracellular cell structural components and secreted factors were used in predation experiments to identify structures that influence predation. No change was measured in the ability of the predator to prey on *S. marcescens* flagella, fimbria, surface layer, prodigiosin and phospholipase-A mutants. However, higher predation was measured on *S. marcescens* metalloprotease mutants. Complementation of the metalloprotease gene, *prtS*, into the protease mutant, as well as exogenous addition of purified serralysin metalloprotease, restored predation to wild type levels. Addition of purified serralysin also reduced the ability of *M. aeruginosavorus* to prey on *Escherichia coli*. Incubating *M. aeruginosavorus* with purified metalloprotease was found to not impact predator viability; however, pre-incubating prey, but not the predator, with purified metalloprotease was able to block predation. Finally, using flow cytometry and fluorescent microscopy, we were able to confirm that the ability of the predator to bind to the metalloprotease mutant was higher than that of the metalloprotease producing wild-type. The work presented in this study shows that metalloproteases from *S*. *marcescens* could offer elevated protection from predation.

## Introduction

Predatory prokaryotes are obligatory predators that prey on other bacteria. The most studied predatory bacteria are those from the genus *Bdellovibrio*. However, many other predatory bacteria can be found in nature, one of which includes *Micavibrio*. First isolated from wastewater in 1982^1^, *Micavibrio* spp. are small (~0.5–1.0 μm) curved, Gram-negative, α-proteobacteria bacteria^2^ with a single polar flagellum. In order to grow, *Micavibrio* spp. utilizes an epibiotic life cycle in which free-swimming motile attack cells attach to the cell surface of prey bacteria in a polar or non-polar manner, followed by an extracellular growth phase as it divides via binary fission^3,4^. In 2011, the complete genome sequence of *Micavibrio aeruginosavorus* ARL-13 was published in addition to transcriptome analysis of the attack and attach growth phase^5^. Additional insight regarding the biology of *M. aeruginosavorus* was presented by Pasternak *et al*.^4^ in a study in which the epibiotic predators *M. aeruginosavorus* ARL-13 and strain EPB were compared to an additional epibiotic predator *Bdellovibrio exovorus* JSS and two periplasmic predators *Bdellovibrio bacteriovorus* HD100 and *B. marinus* SJ. Further studies focused on the prey range of *M. aeruginosavorus*^6–9^, its potential use as a live antibiotic to control infection^10^, its ability to prey on drug resistant pathogens and biofilms^6,11,12^, and the predator’s non-toxic attributes when exposed to mammalian cells lines *in vitro*^8,13^. Additional studies demonstrated the predators’ non-pathogenic characteristics using several animal infection models including rabbit eye wound infection models^14^, mice and rat intranasal and intravenous inoculation models^15–17^, and gastrointestinal inoculation models used to measure the impact on gut bacterial microbiota^18^.

Although our knowledge of the biology, genetics, and potential antimicrobial use of *Micavibrio* has increased considerably in the last few years, the mechanisms governing predation and prey-predator interactions are not yet well understood. In order to prey, *Micavibrio* needs to attach reversibly and then irreversibly to its prey as well as withstand any secreted metabolites or ‘virulence/antimicrobial factors’ produced by the prey cell. In this study, we have used *Serratia marcescens*, an opportunistic human pathogen well studied for its virulence factors^19–21^, as the model prey to examine prey components that may play a role in predation. By using specific mutants defective in key extracellular cell structures and secreted factors, we have identified secreted metalloproteases from *S. marcescens* as being able to alter predation dynamics by reducing the ability of the predator to attach to the prey without affecting predator viability. Genetic manipulation confirmed the role of the metalloprotease in enhancing predation tolerance. Finally, exogenous addition of purified metalloprotease was able to restore predation tolerance to the *S. marcescens* metalloprotease mutant, as well as providing *Escherichia coli* protection from predation.

## Results

### Effect of prey extracellular cell structures and secreted compounds on predation

**Figure 1:**
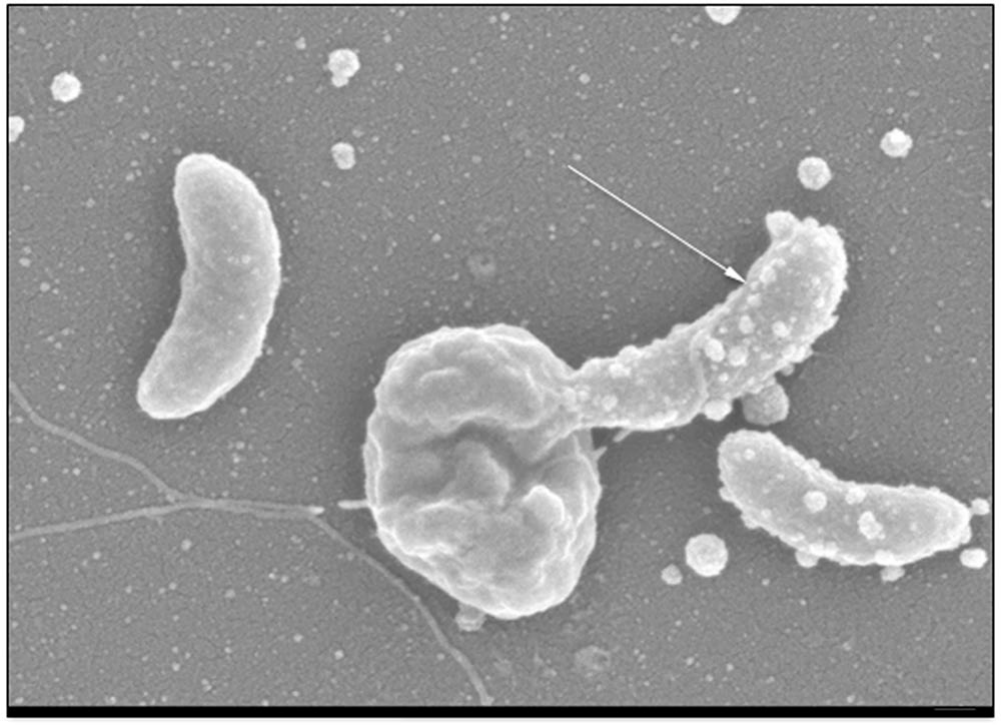
Predation of *M. aeruginosavorus* on *S. marcescens. S. marcescens* wild type K904 was incubated in the presence of *M. aeruginosavorus* for 30 min. The cells were fixed and SEM micrographs were taken. Arrows indicate an attached *M. aeruginosavorus* prey cell to *S. marcescens* prey. Small round spheres are believed to be *S. marcescens* membrane vesicles. Scale bar, 100 nm. Magnification, x50,000.

Extracellular cell structures are the first-thing that the predator encounters as it reaches the prey, it can potentially serve as an attachment site or an obstacle for binding. In this study we have used *S. marcescens* K904 as a model prey. An initial experiment confirmed that when co-cultured, *M. aeruginosavorus* is able to reduce *S. marcescens* K904 viability by 0.30 ± 0.27 and 1.26 ± 0.07 log_10_ within 24 and 48 hrs, respectively (from an initial 1.8 ± 0.3 × 10^9^ CFU/ml to 1.08 ± 0.7 × 10^9^ following 24 hrs and 1 ± 0.01 × 10^8^ following 48 hrs of predation). No substantial change was measured in *S. marcescens* viability following incubation with predator free control (from an initial 1.8 ± 0.3 × 10^9^ CFU/ml to 3.6 ± 1.1 × 10^9^ and 2.3 ± 2.8 × 10^9^ following a 24 and 48 hrs of predation, respectively). Additional confirmation that *M. aeruginosavorus* is able to attach to K904 was done by SEM imaging (Fig. 1). In order to evaluate if prey extracellular structures play a role in predation by *M. aeruginosavorus*, predation experiments were conducted in which the predator was co-cultured with *S. marcescens* mutants deficient in synthesis of candidate extracellular cell structure components that play a role in motility, attachment and protection from environmental challenges (Table 1). No significant difference (p > 0.1) was measured in the ability of *M. aeruginosavorus* to prey on *S. marcescens* mutants defective in flagella, fimbriae, and surface layer protein production, when compared to predation measured on the *S. marcescens* wild-type background strain (Table 1). In addition to cell extracellular structures, microbial secreted compounds might also influence predation. *S. marcescens* secretes several compounds, which have a role as virulence factors, and having known antimicrobial attributes. As seen in Table 1, no significant difference (p > 0.1) was seen in the ability of *M. aeruginosavorus* to prey on *S. marcescens* mutants defective in the production of the prodigiosin and phospholipase-A, compared to the wild-type background. However, the predation on a metalloprotease deficient mutant was significantly higher (p < 0.001) than that measured for the wild type protease producing isolate, with a 2.8 and 1.5 log_10_ reduction respectively (Table 1). In all experiments, maximum predation reduction was measured at the 48 hr time point (data not show). No reduction was seen in any of the mutants following incubation with predator free control when compared to the initial time point (0.06 ± 0.17 log_10_ reduction at 48 hrs).

**Table 1:** Predation of *S. marcescens* K904 by *M. aeruginosavorus* following 48 hrs of incubation.

Prey/mutants	Log Reduction
Wild type K904	−1.56 ± 0.28
**Extracellular cell structures mutants**	
Flagella *fliC*::pMQ192	−1.78 ± 0.24
S-layer *∆slaA*	−1.77 ± 0.27
Pilli/Fimbrae *fimC*::pMQ167	−1.19 ± 0.53
**Secreted compounds mutants**	
Prodigiosin *∆pigA*	−1.50 ± 0.23
Phospholipase A *phlA*::pMQ215	−0.94 ± 0.22
Triple metalloprotease mutant *∆prtS ∆slpB slpE*	−2.80 ± 0.35^*^

Co-cultures were prepared by adding ∼2 × 10^9^ CFU/ml prey cells to harvested predator cells (∼5 × 10^7^ PFU/ml of *M. aeruginosavorus*) or predator free control (*n* = 9). Values represent the Log_10_ change when compared to the predator free control. Each value represents the mean of three experiments done in triplicates with standard deviation.

**−** = Decrease in host numbers.

*Significant difference from the reduction measured for the WT (p < 0.001, One Way ANOVA with Tukey’s post-test).

### Predation on *S. marcescens* metalloprotease mutants

**Figure 2:**
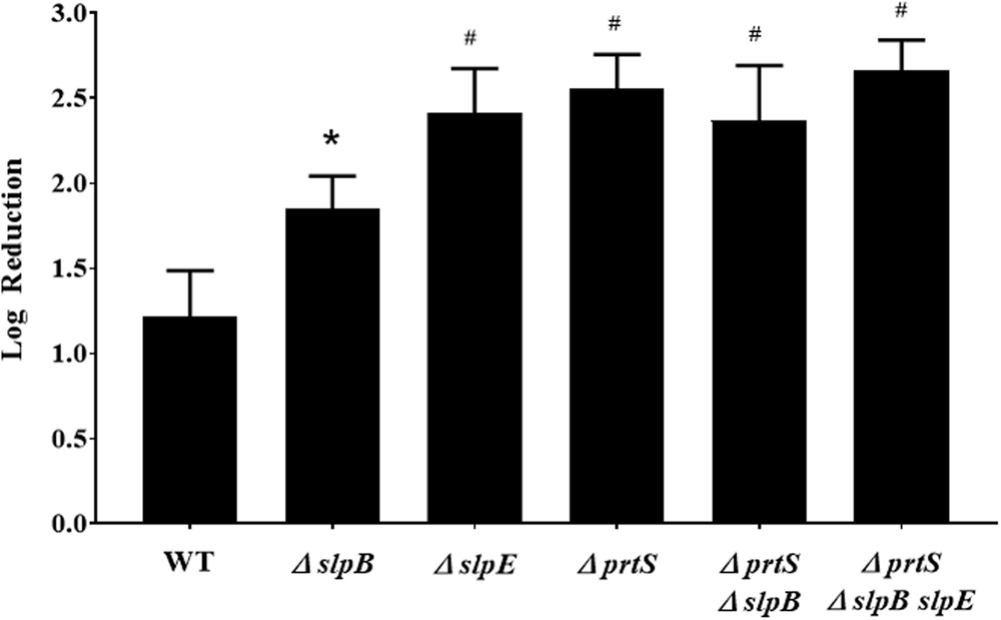
Predation on *S. marcescens* metalloprotease mutants. Co-cultures were prepared by adding *M. aeruginosavorus* to *S. marcescens* wild type (WT) prey, single (∆*prt*S, ∆*slp*B, and *slpE*::pMQ118), double (∆*prt*S ∆*slp*B) or triple (∆*prt*S ∆*slp*B *slpE*) metalloprotease mutants (*n* = 9). Values represent the Log_10_ reduction in prey viability, compared to the predator free control, following 48 hrs of predation. Each value represents the mean and standard deviation of three experiments done in triplicates. Asterisk indicates significant difference from the reduction measured for the WT (p = 0.01). Pound signs indicate significant difference from the reduction measured for the WT (p < 0.001, One Way ANOVA with Tukey’s post-test).

*S*. marcescens strain K904 produces three serralysin family metalloproteases produced by 3 separate genes *prtS*, *slpB*, and *slpE*^22^. To determine which of the three genes play a role in providing *S. marcescens* elevated protection from predation, co-cultures using *S. marcescens* mutants mutated in each of the genes were conducted. As seen in Fig. 2, a slight increase was measured in the ability of the predator to prey on *slpB* compared to the wild type (p = 0.01). However, *prtS*, *slpE* or a double *prtS slpB* mutants showed similar enhanced predation characteristics as the triple *prtS slpB slpE* mutant (Fig. 2), suggesting that the metalloproteases produced by *prtS*, *slpE,* and to a lesser extent *slpB*, were able to provide elevated protection from predation. No differences in cell growth were measured between the wild type and the triple *prtS slpB slpE* mutant, both reaching similar OD’s following 12 hrs of incubation in LB (OD_600_ of 1.6 ± 0.01 and 1.5 ± 0.02 for the wild type and mutant, respectively). Furthermore, the ability of the triple *prtS slpB slpE* mutant to survive in HEPES buffer for 48 hrs was equivalent to that seen in the wild type (from 1.5 × 10^9^ CFU/ ml to 7.0 × 10^8^ CFU/ml for the wild type and from 1.2 × 10^9^ CFU/ ml to 5.8 × 10^8^ CFU/ml for the mutant) (Supplementary material Fig. 1).

**Figure 3:**
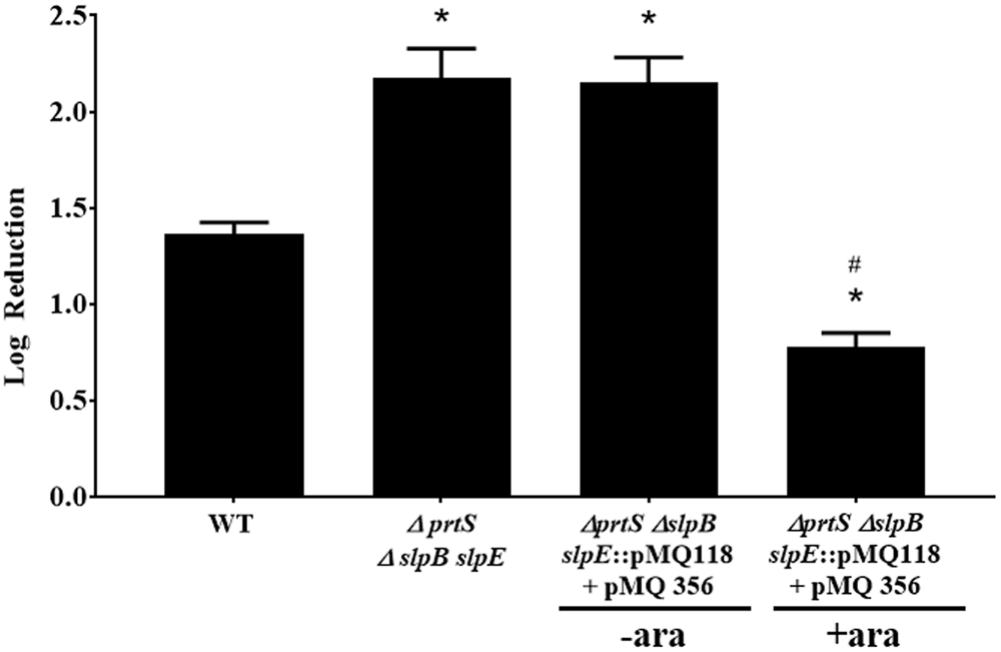
Genetic complementation. Co-cultures were prepared by adding *M. aeruginosavorus* to *S. marcescens* wild- type (WT) prey, triple (∆*prtS* ∆*slpB slpE*) metalloprotease mutants (*n* = 9) or metalloprotease mutants harboring the *prtS* gene under the regulation of an arabinose inducible promoter (∆*prtS* ∆*slpB slpE*::pMQ118 + pMQ356) (*n* = 9). Co-cultures were incubated with or without arabinose (+ara, −ara). Values represent the Log_10_ reduction in prey viability, compared to the predator free control, following 48 hrs of predation. Each value represents the mean and standard deviation of three experiments done in triplicates. Asterisks indicate significant differences from the reduction measured for the WT (p < 0.001). Pound signs indicate significant difference from the reduction measured for the arabinose-free triple metalloprotease mutants (p < 0.001, One Way ANOVA with Tukey’s post-test).

To further validate that the metalloproteases could provide elevated protection from predation, the *prtS* gene was introduced into the triple metalloprotease deficient mutant under the regulation of an inducible promoter using plasmid pMQ356. As before, the ability of *Micavibrio* to prey on the wild type metalloprotease producing *S. marcescens* was reduced compared to that seen on the metalloprotease negative mutant, with 1.3 and 2.3 log_10_ reduction respectively (Fig. 3). Complementation of the *prtS* gene into the metalloprotease mutant, under non-inducing conditions, did not alter predation (2.15 log_10_ reduction, p > 0.1). However, inducing the *prtS* construct with arabinose complemented the mutant defect and reduced predation to levels that are lower than that measured on the metalloprotease producing wild type (0.78 log_10_ p < 0.001), and significantly lower (p < 0.001) to that measured on the metalloprotease mutant (Fig. 3, ∆*prtS* ∆*slpB slpE*). No change in wild type predation was measured in an arabinose treated co-culture control indicating that the arabinose inducer itself was not responsible for the effect (data not shown).

### Purified serralysin metalloprotease from *S. marcescens* can protect prey from predation by *M. aeruginosavorus*

**Figure 4:**
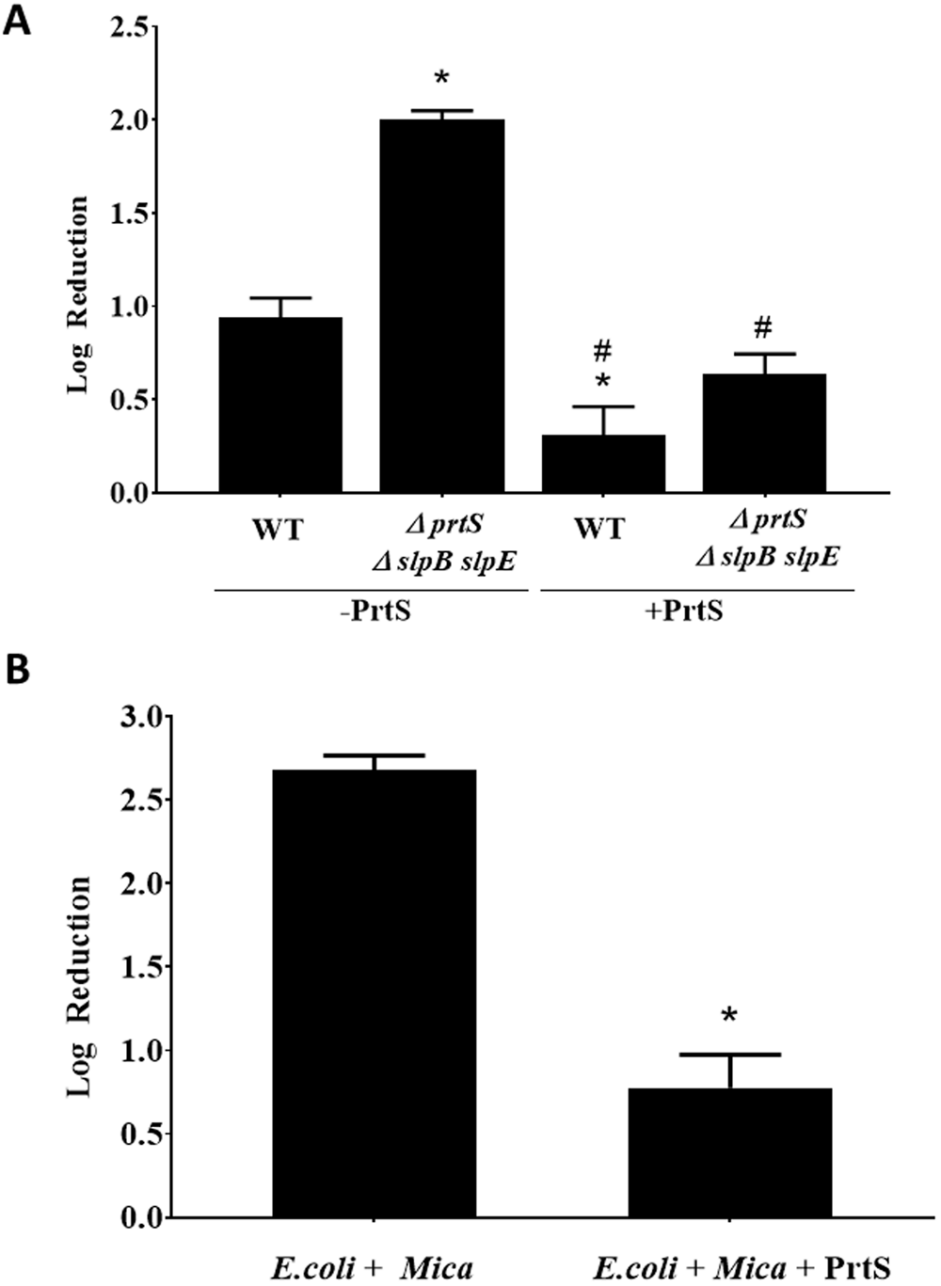
Exogenous addition of purified metalloprotease. (**A**) Co-cultures were prepared by adding *M. aeruginosavorus* to *S. marcescens* wild-type (WT) prey, or triple (∆*prtS* ∆*slpB slpE*) metalloprotease mutants and incubated with or without the addition of purified metalloprotease at 5 µmol/ml final concentration (+PrtS, −PrtS) (*n* = 9). (**B**) Cultures were prepared by adding *M. aeruginosavorus* to *E. coli* (*E.coli* + *Mica*) (*n* = 9) or *E. coli*, with predator and purified metalloprotease (*E.coli* + *Mica* + PrtS) (*n* = 9). Values represent the Log_10_ reduction in prey viability, compared to the predator free control, following 48 hrs of predation. Each value represents the mean and standard deviation of three experiments done in triplicates. Asterisks indicate significant differences from the reduction measured for the WT (A) or *E. coli* + *Mica* (**B**) (p < 0.001). Pound signs indicate significant difference from the reduction measured for the arabinose-free triple metalloprotease mutants (p < 0.001, One Way ANOVA with Tukey’s post-test).

As endogenous production of metalloproteases by *S. marcescens* was shown to reduce predation, we were interested in examining if exogenous metalloprotease was sufficient to reduce predation. To this end, the PrtS metalloprotease (serralysin) from *S. marcescens* was purified and added to predator co-cultures. Adding 5 µmol/ml (final concentration) of pure PrtS to the metalloprotease mutant reduced the ability of the predator to prey to levels lower than those measured for the wild type (0.64 and 1.18 log_10_ predation reduction, respectively) (Fig. 4A). Purified PrtS was also able to render the wild- type strain almost resistant to predation with only a 0.3 ± 0.15 log_10_ reduction following 48 hrs of incubation (Fig. 3A, WT + PrtS). Similar predation protection was seen when purified exogenous PrtS was added to a non-producing metalloprotease *E. coli.* Predation was reduced from 2.6 ± 0.09 log_10_ seen on protease free control (Fig. 4B, *E. coli* + *Mica*) to 0.78 ± 0.19 log_10_ on the *E. coli* protease treated co-cultures (Fig. 4B, *E. coli* + *Mica* + PrtS). Adding purified PrtS to predator-free prey cells did not bring about reduction in *E. coli* cell viability (from an initial 4.5 × 10^9^ CFU/ml to 5 × 10^9^ following 48 hrs of incubation for *S. marcescens* and from an initial 2 × 10^9^ CFU/ml to 1.8 × 10^9^ for *E. coli*). To examine if other proteases have similar predation enhancement effect as metalloproteases, proteinase K and trypsin were added to predator prey co-cultures. No difference in predation was measured when 100 µg/ml (final concentration) proteinase K and 50 µg/ml (final concentration) trypsin was added to a predator co-culture using *S. marcescens* metalloprotease mutant, with a 2.5 log_10_, 2.7 log_10_, and 2.4 log_10_ predation reduction measured for the metalloprotease mutant alone, with proteinase K or trypsin, respectively.

### Serralysin metalloprotease does not impact predator viability

One mechanism in which *S. marcescens* metalloproteases could reduce predation is by toxicity to the predator. To this end, *M. aeruginosavorus* was incubated for 48 hrs in HEPES buffer or HEPES treated with 5 µmol/ml purified PrtS metalloprotease. No reduction was measured in the viability of the predator incubated with the protease compared to that of the protease free control (from an initial 1.0 × 10^8^ PFU/ml to 1.3 × 10^8^ and 1.2 × 10^8^ following 48 hrs of incubation with protease and protease free control, respectively).

### Serralysin metalloprotease impacts attachment to prey cell

**Figure 5:**
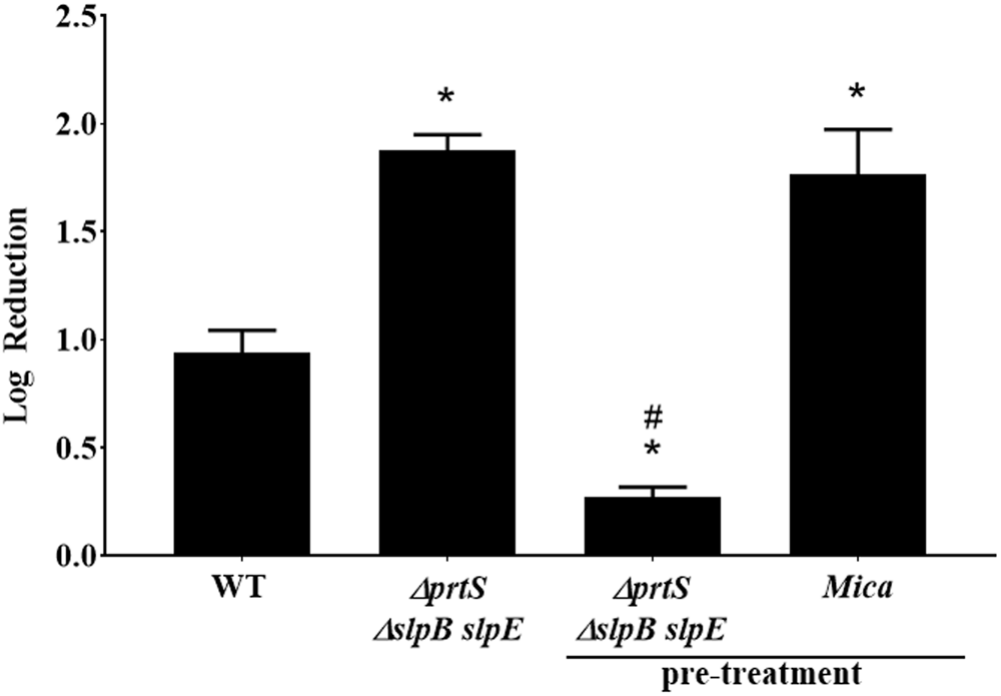
Pre-incubation with purified metalloprotease. Co-cultures were prepared by adding *M. aeruginosavorus* to *S. marcescens* wild-type (WT) prey, or triple (∆*prtS* ∆*slpB slpE*) metalloprotease mutants. Additional co-cultures included the triple metalloprotease mutants that were pre-incubated for 4 hrs with 5 µmol/ml final concentration of PrtS before being washed and added to the culture (∆*prtS* ∆*slpB slpE*-pre-treat) (*n* = 9) or *M. aeruginosavorus* that was pre-incubated for 4 hrs with PrtS before being washed and added to the culture (Mica-pre-treat) (*n* = 9). Values represent the Log_10_ reduction in prey viability, compared to the predator free control, following 48 hrs of predation. Each value represents the mean and standard deviation of three experiments done in triplicates. Asterisks indicate significant differences from the reduction measured for the WT (p < 0.001). Pound signs indicate significant difference from the reduction measured for the arabinose-free triple metalloprotease mutants (p < 0.001, One Way ANOVA with Tukey’s post-test).

A second potential mechanism is that metalloproteases may affect attachment and binding by potentially impacting elements on the surface of the prey or the predator. Protease pre-treatment experiments were conducted in which *S. marcescens* triple metalloprotease mutant or *M. aeruginosavorus* were pre-treated for 4 hrs with 5 µmol/ml purified PrtS. The protease was removed, and the cells were used in a predator-prey co-culture. As before, *M. aeruginosavorus* was able to prey better on the metalloprotease mutant than on the wild type (Fig. 5, ∆*prtS* ∆*slpB slpE*). Pre-incubation of the predator (Fig. 5, *Mica*-pre-treat) with purified metalloprotease did not inhibit the ability of the predator to prey, resulting in similar prey reduction to that measured for the metalloprotease control (1.7 ± 0.2 and 1.8 ± 0.07 log_10_ predation reduction, respectively). However, pre-incubation of the metalloprotease mutant with PrtS (Fig. 5, ∆*prtS* ∆*slpB slpE*-pre-treat) had reduced the ability of the predator to prey, resulting in a 0.27 ± 0.04 log_10_ reduction after 48 hrs. As before, incubating the predator or prey for 4 hrs with the metalloprotease did not reduce cell viability with the predator numbers remaining at 1 × 10^8^ PFU/ml. Our data suggests that metalloprotease may impact predation by affecting the prey, while transmission electron microscopy (TEM) did not provide additional insight regarding any specific structures that may differ from the wild type and metalloprotease mutant. However, some changes in cell wall surface are evident in the TEM micrographs with what seems as a denser and opaquer cell wall structure in the metalloprotease mutant compared to the wild type (Supplementary material Fig. 2). No difference in yeast agglutination was seen between the wild type and metalloprotease mutant with aggregation occurring 20 seconds after mixing bacteria and yeast cells. Whereas, PBS (no bacteria) control and fimbriae mutant (*fimC*::pMQ167) failed to agglutinate with yeast (Supplementary material Fig. 4C).

**Figure 6:**
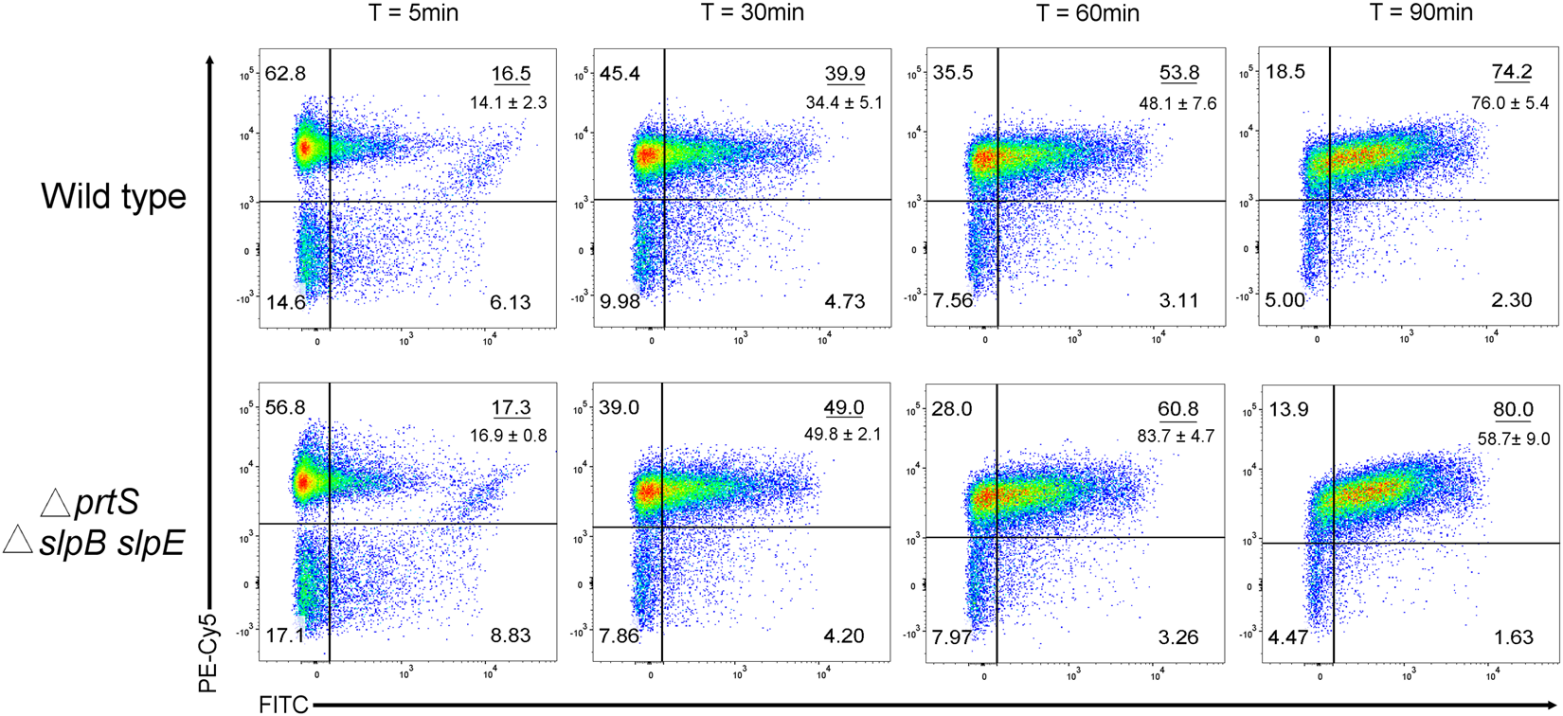
Attachment dynamics of *M. aeruginosavorus*. FM 4–64 stained *S. marcescens* (Wild type) prey or triple (∆*prtS* ∆*slpB slpE*) metalloprotease mutant were co-cultured with PKH67 stained *M. aeruginosavorus* for 5, 30, 60, and 90 min (*n* = 3). Cells were incubated with the dyes for 10 minutes. Cells were then spun down and resuspended in HEPES to remove excess dye. Immediately after, cells were mixed together for 5, 30, 60, and 90 minutes. The top right number is a representative value of the percent population of *M. aeruginosavorus* attached to either wild type or triple metalloprotease mutant. The number below represents the average of three independent experiments. Analysis of the data was done on FlowJo v10.4.

**Figure 7:**
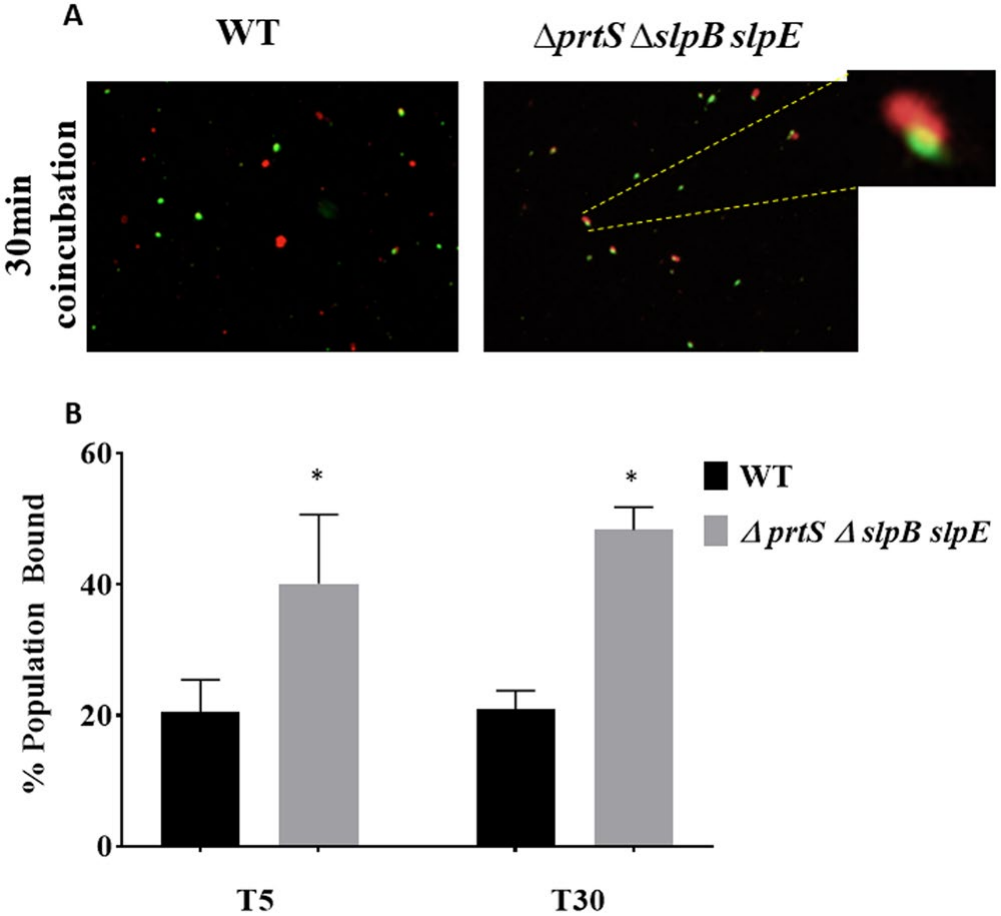
Attachment of *M. aeruginosavorus*. (**A**) FM 4–64 stained *S. marcescens* (Wild type) prey or triple metalloprotease mutant (∆*prtS* ∆*slpB slpE)* were co-cultured with PKH67 stained *M. aeruginosavorus*. Cells were incubated for 30 min before being fixed in 1% formaldehyde and examined using a fluorescent microscope with 40x digital zoom and 40x magnification. Predator cells are seen in green, while prey cells seen are in red. The image in the upper right corner represents digital magnification of *Micavibrio*-*Serratia* binding. Celena-S and Microsoft PowerPoint software were used for image brightness adjustment and digital magnification. Experiments were conducted 3 times with representative images shown. (**B**) Attachment of *S. marcescens* wild type prey or triple metalloprotease mutant following 5 and 30 min of incubation as visualized using Celena-S digital fluorescence microscope with GFP and DAPI filters. Bars represent mean and standard deviation of percent of bound *M. aeruginosavorus* to *S. marcescens* calculated from total predator numbers in a field of view. The experiment was performed three times with 6 fields measured for each treatment. Asterisks indicate significant differences from attachment measured for the WT (p = 0.005).

In order to further measure the impact of metalloproteases on predator binding, flow cytometry was used to evaluate initial *M. aeruginosavorus* - *S. marcescens* cell-cell attachment. Prey and predator cells were stained, mixed and incubated together for 5 to 90 min. Predator cell association was found to be time dependent with an average of 14%, 34%, 48% and 76% prey attached to the wild type at the 5, 30, 60 and 90 min, respectively. However, the affinity of the predator to bind to the metalloprotease mutant was higher than that measured for the wild type with an average of 17%, 50%, 59% and 84% prey attached at the 5, 30, 60 and 90 min, respectively. Maximum binding difference was seen at the 30 min time point with 47% more cells attached to the mutant than that of the wild type (p = 0.04) (Fig. 6). The enhanced ability of the predator to bind to the metalloprotease mutant was also confirmed by fluorescence microscopy (Fig. 7A). An average of 20.5 ± 4.9% and 21 ± 2.7% of prey cells were found to be attached to the wild type after the 5 and 30 min of co-incubation, respectively. Whereas an average of 40.1 ± 10.5% and 48.3 ± 3.5% of the predator were found to be attached to the metalloprotease mutant at the same time points (p = 0.005) (Fig. 7B).

## Discussion

In order to feed, survive and proliferate, the epibiotic predator *M. aeruginosavorus* needs to get in close proximity to its prey before adhering to the prey surface. Thus, we speculated that prey secreted compounds and or surface structures may play a role in predation as inhibiting factors or as facilitators of attachment. In a 2007 study we were unsuccessful in isolating *P. aeruginosa* PA14 predation-resistant mutants in a screen using a random transposon library^6^. It was suggested, that prey structures that play a role in predation might be essential, therefore, making them unable to be found in a library of viable mutants. Another explanation for the inability to isolate prey resistant transposon mutants is that genes or pathways required for predator-prey interactions are redundant. In this study, we have used a more direct approach in which specific deletion mutants of *S. marcescens* K904 were used. *S. marcescens* is emerging as an important opportunistic pathogen^20^. Many of the mutants used to study *S. marcescens* virulence are cell structural components and secreted factors that might also play a role in predation. Although we have previously reported that *M. aeruginosavorus* ARL-13 is unable to prey on *S. marcescens* D 217 and PIC3611/371^6,7^, here we report that *M. aeruginosavorus* ARL-13 is able to prey on *S. marcescens* K904. SEM imaging (Fig. 1) and prey enumeration following predation confirmed predator attachment and predation. As previously seen with other prey, maximum prey cell reduction is measured following 48 hrs of predation^7,8^. Differences in strain specificity and prey susceptibility are well established for both *M. aeruginosavorus* and *Bdellovibrio*^6–8^. Thus, it is not uncommon to see predators preying on one strain within a given species and not another.

Deletion of fimbria, which plays a role in cell-cell and cell-surface association^23–25^, did not impact predation. Thus, it could be concluded that fimbria does not serve as an attachment site nor could it interfere in the ability of the predator to reach any putative prey binding sites. Elimination of flagella biosynthesis did not impact predation, as well, suggesting that flagellin does not serve as a predator-binding site. Although the motility of the prey might alter predator-prey encounter, the prey to predator ratio, as well as the small liquid volume used in the study is likely to allow sufficient random collisions of predator and prey, regardless of prey motility^26,27^. Koval and Hynes suggested that the paracrystalline protein surface layer (S-layer) of *Aquaspirillum serpens, A. sinuosum* and *Aeromonas salmonicida* provides protection from *B. bacteriovorus* predation^28^. Here we found no significant effect in the ability of *Micavibrio* to prey on *S. marcescens* S-layer mutants. Although we confirmed that the *slaA* mutant does not produce S-layer protein and exhibits a visually different cell wall structure (Supplementary Fig. 3), we could not determine if the S-layer evenly coats the entire cell surface of the wild type. The ability of *Micavibrio* to easily penetrate the thick extracellular polymeric substances produced by biofilms^6,7^ may suggest that the S-layer is not sufficient to block predation. Although we have selected several mutants that were deficient in key cell surface structures, future work needs to be conducted in order to evaluate additional structures that may play a role in predation such as bacterial capsule and lipopolysaccharides^29^. Before encountering the prey, predators can come across a variety of prey secreted proteins, enzymes, lipids, biosurfactants as well as other antimicrobials that might impact predation. Indole, for example, was shown to impact predation of *E. coli* and *Salmonella enterica* by *B. bacteriovorus*^30^. Medium acidification, in diluted nutrient broth but not HEPES buffer, a byproduct of prey sugar metabolism, was also shown to block predation. The effect was seen in co-cultures using *B. bacteriovorus* and *M. aeruginosavorus*, and was attributed to the inability of the predator to survive in acidified environments^31^. *S. marcescens* was shown to produce an arsenal of compounds which likely play a role in microbial pathogenesis, among them are phospholipase-A^32^, proteases^33^, as well as prodigiosin^34,35^ which has antimicrobial attributes, as well. In this study, we have found no change in the ability of *M. aeruginosavorus* to prey on *S. marcescens* mutants defective in the production of prodigiosin and phospholipase-A. This finding is in accord with the fact that *M. aeruginosavorus* is able to attack other human pathogens, which are known to secrete a variety of toxins and antimicrobials, such as *Burkholderia mallei, P. aeruginosa* and *Yersinia pestis*^6–9,11^. An additional virulence factor that is produced by *S. marcescens* K904 are metalloproteases which are members of the RTX-toxin family^36,37^ and were shown to be cytotoxic to mammalian cells *in vitro*^36,38,39^. Although phospholipase mutant still showed some lipase activity (Supplementary material Fig. 6), the *S. marcescens* genome harbors numerous predicted lipases and esterase genes that may be active in the lipase assay.

In this study, we confirmed that metalloprotease producing strain *S. marcescens* K904 was less susceptible to predation than the metalloprotease deficient mutants. Predation protection was found to be mediated by each of the metalloprotease producing genes (Fig. 2). Interestingly, *slpB*, which showed reduced predation protection capability (Fig. 2), was also shown to produce lower levels of metalloprotease compared to *prtS* and *slpE*^39^. Therefore, metalloprotease predation protection capability may be dose dependent. The enhanced resistance of the wild type following the addition of exogenous purified PrtS also supports the conclusion that metalloprotease predation resistant attributes may be dose dependent (Fig. 4A). Genetic complementation and the ability of purified PrtS to restore predation of the hyper-susceptible triple *prtS slpB slpE* mutant to wild type levels validated the role of metalloprotease in enhancing resistance to predation. The predation protection effect of metalloprotease was also confirmed by adding purified PrtS to *E. coli* (Fig. 4B). The resistance conferring activity of the metalloprotease was found to be specific and was not seen when using proteinase K or trypsin that, unlike PrtS, did not alter predation. The ability of proteases to block predation was previously reported in a study that demonstrated that the addition of proteinase K but not trypsin or saliva, which is known to harbor trypsin-like proteases, could block predation of *E. coli* and *Aggregatibacter actinomycetemcomitans* by *B. bacteriovorus*. It suggested that proteinase K may affect specific surface proteins on the prey cell or the *B. bacteriovorus* that are required for predation^40^. Although metalloprotease provided enhanced protection from predation by *M. aeruginosavorus* it did not seem to provide protection from predation by *B. bacteriovorus* (Supplementary Table 1).

Incubating *M. aeruginosavorus* with purified PrtS was found not to impact predator viability; however, pre-incubating the prey, but not the predator, with purified metalloprotease was able to block predation (Fig. 5). It could be hypothesized that metalloprotease may affect or modify cell surface components that facilitate predation, causing reduced predation. TEM micrographs showed some changes in the thickness of the cell wall of the wild type compared to the metalloprotease mutant. However, with the limited resolution of TEM and its inability to detect changes that might involve smaller structures such as protein receptors, it is difficult to make any assumptions regarding modified specific structures and their impact on predation. Nonetheless, flow cytometry and microscopy evaluation confirmed that the ability of the predator to bind to the metalloprotease mutant was higher than that of the metalloprotease producing wild type (Figs 6 and 7A,B). No difference in yeast agglutination, a phenotype associated with fimbriae production, was measured between the wild type and the metalloprotease mutant (Supplementary material Fig. 4C). Several species of bacteria such as *Erwinia*^41^, which was found not to be preyed upon by *M. aeruginosavorus*^7^ and *Serratia*^22^ carry up to five highly similar metalloprotease genes in their genomes. Given the functional overlap of the proteins, it is not clear what drives selection to maintain these seemingly redundant proteins. Based on this study, it is tempting to speculate that the one driving force in maintaining these genes is the protection they provide from predatory prokaryotes such as *Micavibrio*.

In conclusion, the work presented in this study shows that metalloproteases from *S. marcescens* offer prey elevated protection from *Micavibrio* predation. This is the first report, to our knowledge, of a mechanism that prey use in order to defend themselves against *Micavibrio*. This study also offers additional insights on prey factors that may play a role in *M. aeruginosavorus* prey interaction and prey specificity. Future studies will focus on better understanding the specific prey components that are impacted by metalloprotease and to determine whether metalloproteases from other bacterial prey species provide protection from predation.

## Methods

### Bacterial strains and growth conditions

The *S. marcescens* used in the study are listed in Table 2. *S. marcescens* were grown routinely in 15 ml lysogeny broth (LB) medium in a 25 ml Polystyrene tissue culture flask at 30 °C. When appropriate, gentamicin and kanamycin were used at 10 µg ml^−1^. Arabinose, which induces expression of *P*_*BAD*_ promoter in pMQ356, was added to the culture (0.02% w/v final concentration). Proteolytic activity of *S. marcescens* was confirmed by a zone of clearance after incubating the bacteria on LB-agar plates containing 2% w/v skim milk. *Escherichia coli* strain ST-2289, a multi drug resistant KPC-3 isolate^42^, was kindly provided by Dr. Kreiswirth of Rutgers, New Jersey Medical School, and cultured in LB at 37 °C. *M. aeruginosavorus* strain ARL-13 was cultured and maintained as described previously^7,8,11^, using *E. coli* strain WM3064 as prey. Predator stock-lysates were made by co-culturing the predators with prey cells in HEPES buffer (25 mM) supplemented with 2 mM CaCl_2_ and 3 mM MgCl_2_. Co-cultures were incubated at 30 °C for 72 hrs, until the culture cleared (stock-lysates). Stock-lysates were filtered through a 0.45-µm Millex pore-size filter (Millipore) to remove any remaining prey and used in predation experiments (harvested predators). Prey and predator cells were enumerated as colony-forming units (CFU) or plaque forming units (PFU) developing on LB agar plates or lawns of prey cells^6^.

**Table 2:** *S. marcescens* strains and plasmids.

Strain	Description	Source or reference
WT-K904	Clinical contact lens-associated keratitis strain	^49^
K904 *fliC*::pMQ192	K904- Flagella mutant with *fliC* insertion mutation.	^25^
K904 *fimC*::pMQ167	K904- Fimbriae mutant with *fimC* insertion mutation.	^25^
K904 *phlA*::pMQ215	K904- Phospholipase-A with *phlA* insertion mutation.	^50^
K904 ∆*slaA*	K904- S-layer mutant with *slaA* deletion mutation.	This study
K904 ∆*pigA*	K904- Prodigiosin mutant with deletion mutation of *pigA*.	^44^
K904 ∆*prtS* ∆*slpB slpE*	K904- Metalloprotease defective with mutation of all three metalloprotease genes.	^22^
K904 ∆*prtS*	K904- Partial Metalloprotease defective with deletion mutation of *prtS*.	^39^
K904 ∆*slpB*	K904- Partial Metalloprotease defective with deletion mutation of *slpB* protease.	^39^
K904 *slpE*::pMQ118	K904- Partial Metalloprotease defective with insertion mutation of *slpE* - protease reduced	^22^
K904 ∆*prtS* ∆*slpB*	K904- Partial Metalloprotease defective with deletion mutation of *prtS* and *slpB*.	^39^
K904 ∆*prtS* ∆*slpB slpE*::pMQ118 + pMQ356	K904 Metalloprotease triple mutant with wild type *prtS* on an inducible plasmid.	^22^

### Cloning and Mutagenesis

To generate the *slaA* mutant, the 3003 base pair open reading frame was [amplified with primers #3743 5′-aaattctgttttatcagaccgcttctgcgttctgatttaCGCGTAGTGCAGAGTATCCAC-3′ and #3744 5′-aattgtgagcggataacaatttcacacaggaaacagctATGTCATCTCTTGTCTCACAGC-3′ using high fidelity polymerase Phusion (New England Biolabs) and] cloned into allelic replacement vector pMQ460^39^ using yeast homologous recombination^43^ and digested with KpnI which removes a *slaA* internal 1482 base pair fragment. The resulting pMQ460 + ∆*slaA* plasmid (pMQ566) was used for allelic replacement of the wild type *slaA* gene as previously described^43^ and the mutant allele on the chromosome was verified by PCR and sequencing.

Phenotypic analysis was performed to demonstrate that secreted and surface components were made by the wild type bacteria and not made by the mutant bacteria under the conditions used for predation analysis. The presence and lack of surface layer protein in wild type K904 and *slaA* mutant, respectively, were determined and confirmed by SDS-PAGE analysis as previously described^44^ (Supplementary material Fig. 3B). Furthermore, transmission electron microscopy (TEM) images demonstrate a change in cell wall structure in the ∆*slaA* mutant consistent with lack of the S-layer (Supplementary material Fig. 3B,C). TEM was also used to confirm the absence of flagella in the *fimC* mutant and presence of flagella in the wild type (Supplementary material Fig. 4A,B). Cells were counted and 0 out of 79 *fimC* mutant cells had flagella, whereas 36.8% (14/38) of the wild type had flagella, p < 0.0001 two-tailed Fisher Exact Test. Yeast agglutination assay was conducted in order to conform the inability of the fimbriae mutant (*fimC*) to bind to yeast (Supplementary material Fig. 4C), a phenomenon that requires fimbriae^45^. The absence of prodigiosin (red pigment) in the ∆*pigA* mutant and production by the wild type under predation experimental conditions was verified visually (Supplementary material Fig. 5). Reduction in phospholipase activity in phospholipase A mutant was confirmed using EnzChek Phospholipase A1 Assay-Kit (ThermoFisher Scientific E1) (Supplementary material Fig. 6).

### Predation Experiments

Predation experiments were performed as described previously with some modifications^7,8,11^. Co-cultures of predator and prey were prepared in 14 ml Falcon™ round-bottom polypropylene tubes by adding 0.4 ml of harvested predators (∼5 × 10^7^ PFU/ml) to 0.4 ml of HEPES washed prey cells (∼2 × 10^9^ CFU/ml) and 1.2 ml HEPES medium. Co-cultures were incubated at 30 °C on a rotary shaker set at 30 r.p.m. Predation was measured by the change in prey population, enumerated by dilution plating, during a 48 hr incubation period. As maximum prey reduction by *M. aeruginosavorus* occurs following 2 days of incubation (see result section), all predation experiments were conducted and reported for the 48 hr time point. Experiments were done at least three times in triplicates. Experiments requiring the addition of purified metalloprotease were conducted twice in triplicates. Phenotypic characterization of prey cells under predation conditions could be seen in Supplementary Figs 1–6.

### Electron microscopy

For scanning electron microscopy (SEM) *M. aeruginosavorus* ARL-13 was co-cultured with *S. marcescens* as described above. Samples were removed following 30 min of incubation and placed on a Cell-Tak coated glass coverslip. Samples were fixed using 2.5% glutaraldehyde for 1 hr, washed with PBS and post-fixed in aqueous 1% OsO4. Coverslips were dehydrated through a graded series of 30% to 100% ethanol and washed with Hexamethyldisilazane. Samples were sputter coated with 6 nm of gold/palladium (Cressington Auto 108, Cressington, UK). Imaging was done using a JEOL JSM- 6335 F scanning electron microscope (Peabody, MA) at 3 kV with the SEI detector. For transmission electron microscopy (TEM) aliquots of cultures grown overnight were washed and suspended in HEPES buffer and applied to formvar coated grids, treated with uranyl acetate (1%), and imaged using a JEM-1210 electron microscope as previously described^24^.

### Yeast agglutination assay

To measure fimbriae production by both the wild type and triple metalloprotease mutant (∆*prtS* ∆*slpB slpE*), yeast agglutination was measured as previously described^25^. Bacteria were grown overnight in LB and resuspended in HEPES. The OD was set to 1.0. Twenty-five microliters of *S. cerevisiae* (Sigma-Aldrich YSC2) in HEPES buffer (0.2 g/10 ml) was mixed with test bacteria (25 µl), or PBS (25 µl) as a negative control on a glass microscope slide and mixed using a rotary rotator (Barnstead Multipurpose Rotator). The rotary platform was set to half of maximum speed and the time to aggregation was measured using a stopwatch.

### Purification of PrtS

Serralysin was overexpressed in *E. coli* and purified under denaturing conditions as previously described^46^. *E. coli* PrtS was induced with 0.02% w/v arabinose for 4 hrs and cells were then centrifuged and resuspended in 50 mM tris and 150 mM NaCl. Cells were lysed by sonication and lysates were cleared by centrifugation. Inclusion bodies were then resuspended in 50 mM Tris, 150 mM NaCl and 6 M GuHCl, prior to purification on a Ni-NTA column (GE Life Sciences). Fractions containing the protease were eluted using 400 mM imidazole and further purified using sephacryl S200 Hi-Prep gel filtration column (GE Life Sciences). The denatured protein was then stored at 4 °C. Refolding and protein concentration were done as previously described by rapid dilution while residual GuHCl was removed using size exclusion chromatography^47,48^. Protease activity was confirmed using a fluorescent casein substrate (Enzchek, ThermoFisher).

### Flow Cytometry

*Serratia marcescens* wild type and triple protease mutant were grown in LB medium at 30 °C overnight. Cells were centrifuged and resuspended in HEPES buffer. Predator stock-lysates were prepared and filtered as described above. Prey and predator cells were stained with red FM 4–64 (Sigma-Aldrich) and green PKH67 (Invitrogen) dyes at 2 nM and 2 mM final concentrations, respectively, and incubated for 10 min. Thereafter, cells were washed by centrifugation and resuspended in HEPES. Prey and predator cells were mixed and incubated for 5, 30, 60 and 90 min, on a rotary shaker at 30 r.p.m. Samples were acquired on the BD LSRFortessa X-20 flow cytometer on low at an approximate rate of 2500ev/sec. Samples were first gated on *S. marcescens* (FM 4–64 positive) and then measured for binding of *M. aeruginosavorus* (PKH67 intensity). Data was acquired on the FITC channel (530/30) and the PE-Cy5 channel (670/30). FlowJo v10.4 software was used for data analysis.

### Fluorescence microscopy

Predator and prey were prepared and stained with red FM 4–64 green PKH67 as described for flow cytometry. Prey and predator cells were mixed in HEPES buffer, as described for predation assays and incubated for 30 min. Following incubation, the bacteria were fixed with 1% formaldehyde for 10 min and mounted on a poly-L-lysine coated slide. Slides were imaged using a Celena-S Digital Fluorescence Microscope with GFP and DAPI filters, 40x digital zoom and a 40x objective lens. Celena-S and Microsoft PowerPoint software were used for image brightness adjustment and digital magnification.

### Statistical analysis

A one-way analysis of variance (ANOVA) with Tukey’ s post-test was performed using Graphpad Prism 6 software. Statistical significance was set to p < 0.001 with standard deviation (SD) values.

## Electronic supplementary material

Supplementary dataset
