# Supplementary material for: Serralysin family metalloproteases protects Serratia marcescens from predation by the predatory bacteria Micavibrio aeruginosavorus
Source: Sci Rep. 2018 Sep 19;8:14025. doi: 10.1038/s41598-018-32330-4 (PMC6145908; doi:10.1038/s41598-018-32330-4)
Supplement: Supplementary file 1 — Supplementary dataset [file 41598_2018_32330_MOESM1_ESM.pdf]

Supplemental Information for

Serralysin family metalloproteases protects *Serratia marcescens* from predation by the predatory bacteria *Micavibrio aeruginosavorus*

Carlos J. Garcia [1], Androulla Pericleous [1], Mennat Elsayed[1], Michael Tran[1], Shilpi Gupta[1], Jake D. Callaghan[2], Nicholas A. Stella[2], Jonathan M. Franks[3], Patrick H. Thibodeau[4], Robert M. Q. Shanks[2], and Daniel E. Kadouri[1*]

[1]Department of Oral Biology, Rutgers School of Dental Medicine, Newark, NJ 07103, USA

[2]Department of Ophthalmology, Charles T. Campbell Laboratory of Ophthalmic Microbiology, University of Pittsburgh, Pittsburgh, PA 15213, USA

[3]Center for Biologic Imaging, University of Pittsburgh, Pittsburgh, PA 15213, USA

[4]Department of Microbiology and Molecular Genetics, University of Pittsburgh, PA, 15221, USA

[*]**Corresponding author:** Daniel E. Kadouri, Department of Oral Biology, Rutgers School of Dental Medicine, Newark, NJ, USA. E-mail: kadourde@sdm.rutgers.edu

Supplementary table 1.

Predation of *S. marcescens* by *B. bacteriovorus* following 12 hrs of incubation.

**Prey/ mutants**	**Log Reduction**
Wild type K904	-4.06 ± 0.54
Triple metalloprotease mutant<br>Δ*prtS* Δ*slpB slpE*	-4.52 ± 0.55

Co-cultures were prepared by adding ~$2x10^9$ CFU/ml prey cells to harvested predator cells (~$5x10^8$ PFU/ml of *B. bacteriovorus*) or predator free control. Values represent the Log_{10} change as compared to the predator free control. Each value represents the mean of three experiments done in triplicates.

- = Decrease in host numbers.

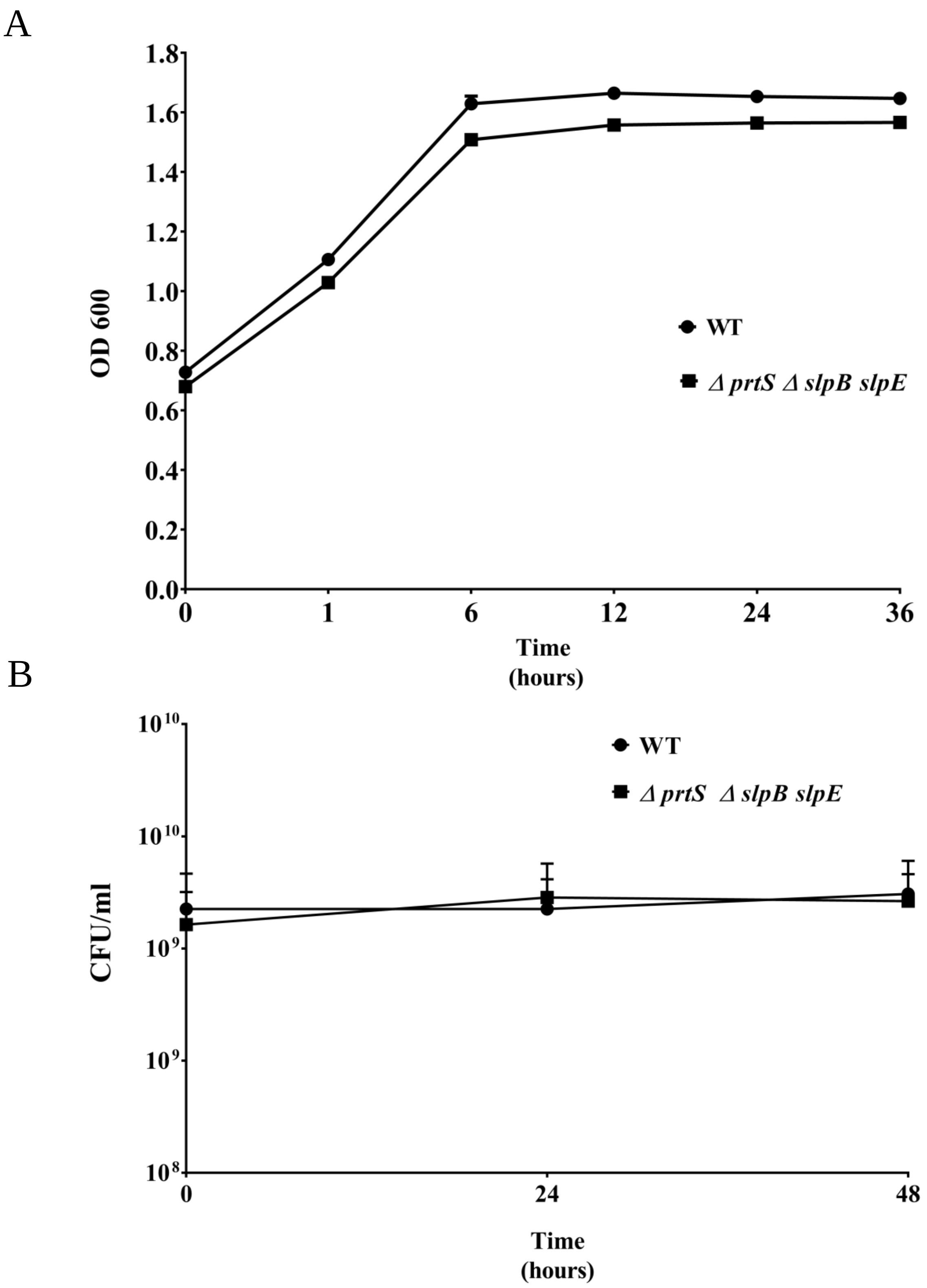

Supplementary Fig 1.

Growth and survival of *S. marcescens*. (A) *S. marcescens* wild type (WT) and triple metalloprotease mutants (Δ*prtS* Δ*slpB slpE*) were grown in LB, growth was measured at OD_{600} (n = 12). **(B)** Cells were grown overnight in LB, washed and resuspended in HEPES buffer. Cell survival was measured by cell enumeration (n = 8).

Wild type   ΔprtS ΔslpB slpE

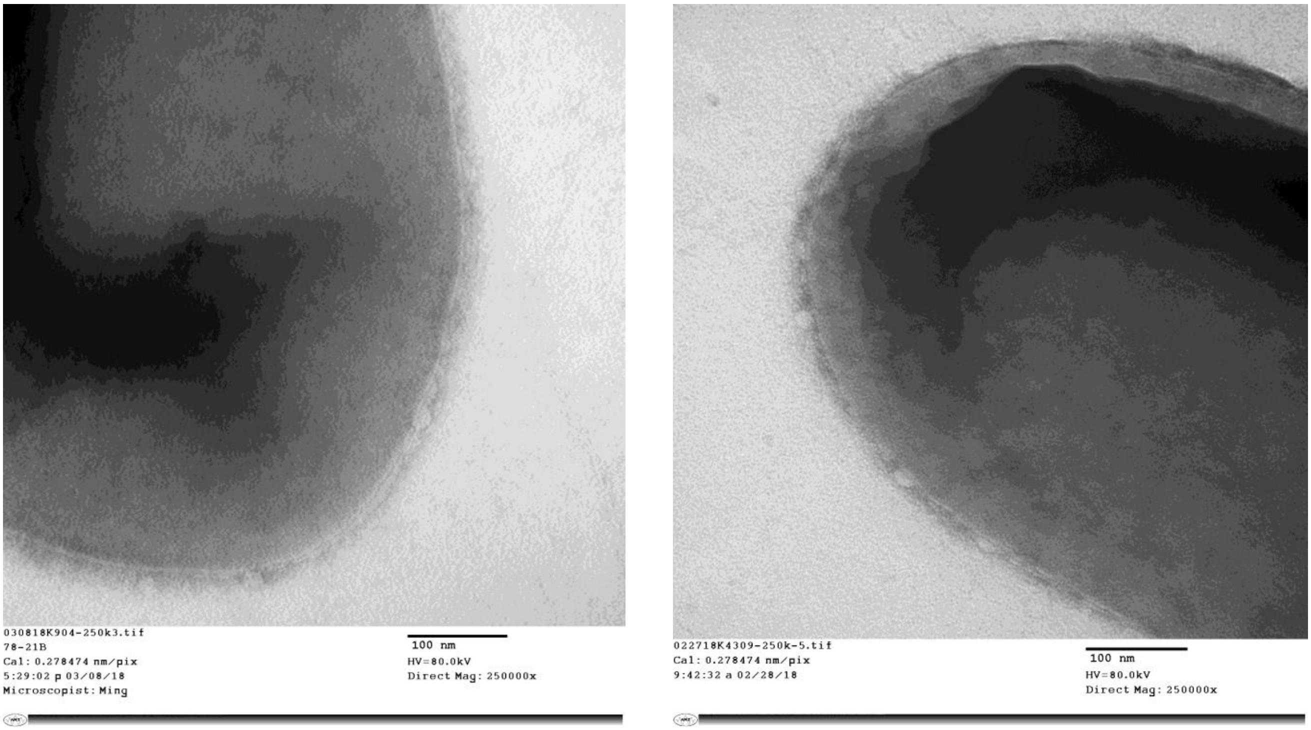

Supplementary Fig 2.

TEM micrographs of *S. marcescens* wild type and the metalloprotease mutant.

S. marcescens wild type and triple metalloprotease mutants (Δ*prtS* Δ*slpB slpE*) were grown in LB, washed in HEPES buffer and taken for TEM imaging. Scale bar, 100 nm. Magnification, x250,000.

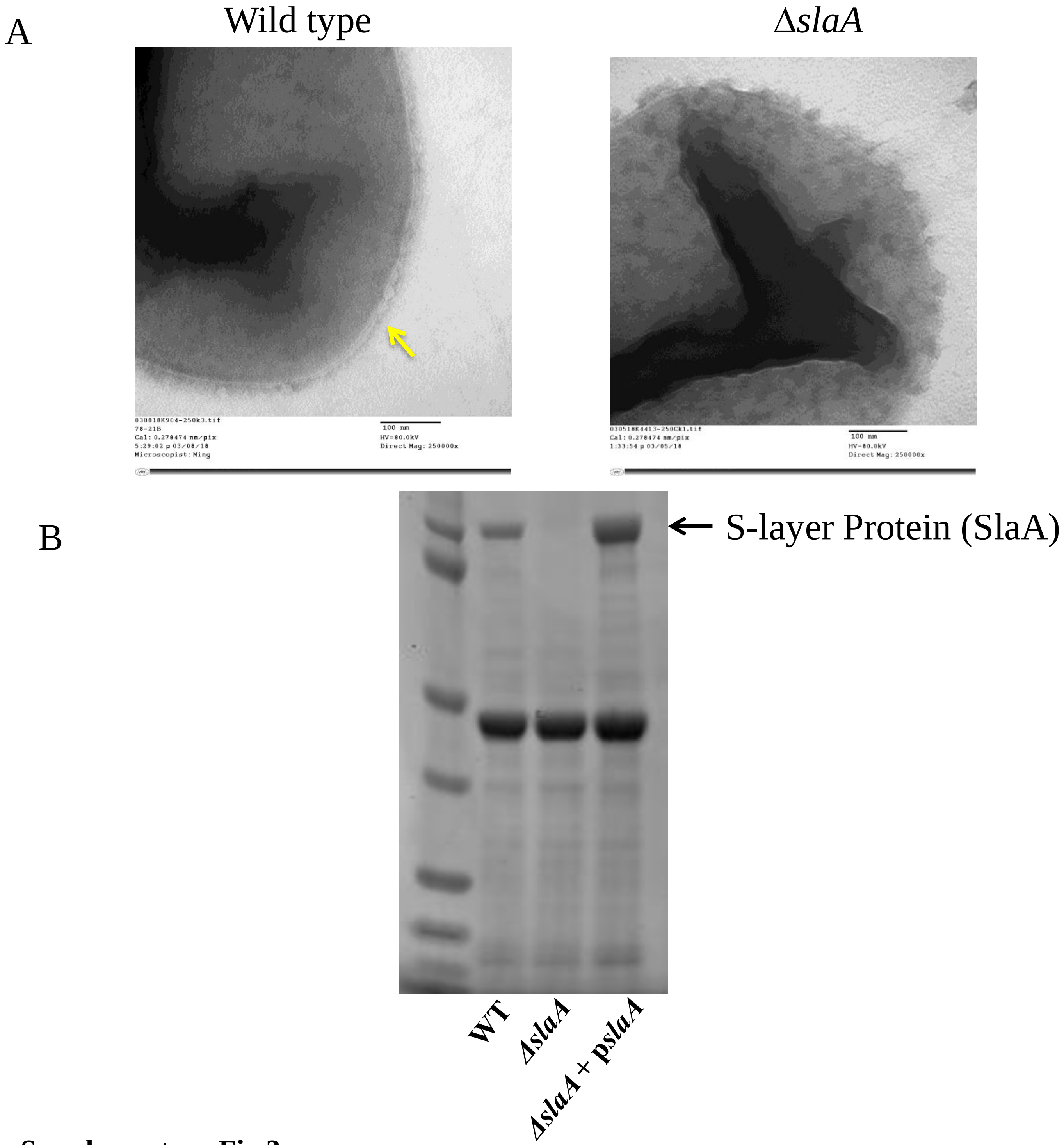

Supplementary Fig 3.

Conformation of S-layer mutation. (A) *S. marcescens* wild type and S-layer mutants (Δ*slaA*) were grown in LB, washed in HEPES buffer and taken for TEM imaging. Scale bar, 100 nm. Magnification, x250,000. Yellow arrow indicates a structure that may be the S-layer. **(B)** SDS-PAGE analysis of *S. marcescens* wild type, S-layer (Δ*slaA*) and a complementation of Δ*slaA* in which plasmid pMQ562 harboring *slaA* was introduced to Δ*slaA*. Cells were grown in LB, washed in HEPES buffer. Surface proteins were isolated, separated on a SDS-PAGE, stained with Coomassie, and imaged with a Licor Odyssey infrared imager. A band corresponding to the S-layer was absent in the mutant and present in the wild type and complemented strain.

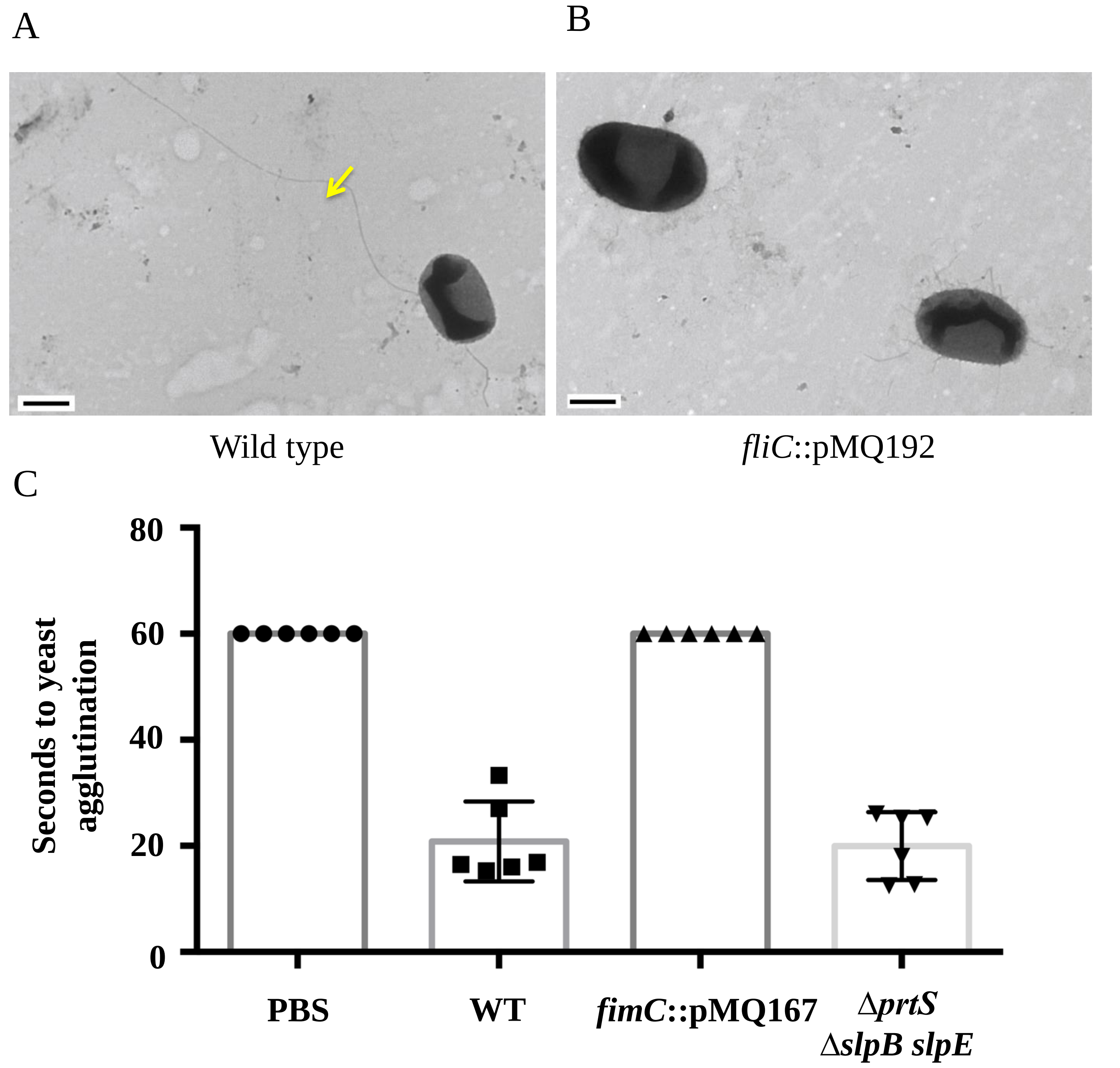

Supplementary Fig 4.
Confirmation of flagella and fimbriae mutant defects. *S. marcescens* K904 wild type **(A)** and flagella mutant (*fliC*::pMQ192) **(B)** were grown in LB, washed in HEPES buffer and taken for TEM imaging. Scale bar, 500 nm. Magnification, x15,000. Yellow arrow indicates a flagellum. **(C)** Fimbriae-dependent yeast agglutination assay. *S. marcescens* wild type and Fimbriae mutant (*fimC*::pMQ167) were grown in LB, washed in HEPES buffer and the time to agglutinate yeast was recorded, to a maximum of 60 seconds. Phosphate buffered saline (PBS) was used as a negative control. The *fimC* mutant, like PBS, failed to agglutinate yeast within 60 seconds. The mean and standard deviation of three independent experiments is shown for each group (n = 6). There was no significant difference between the wild type and the triple protease mutant ($p > 0.05$, ANOVA with Tukey's post-test).

A

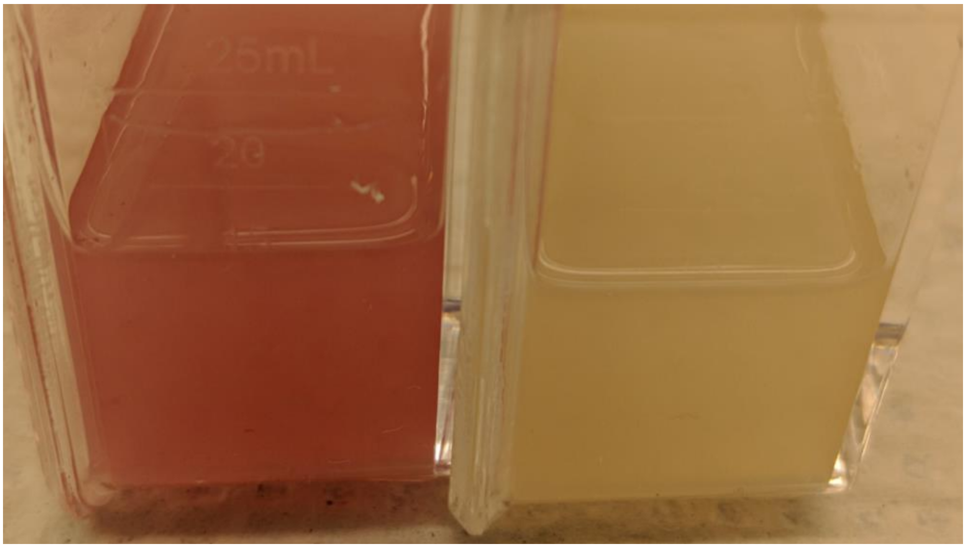

Wild type ∆*pigA*

B

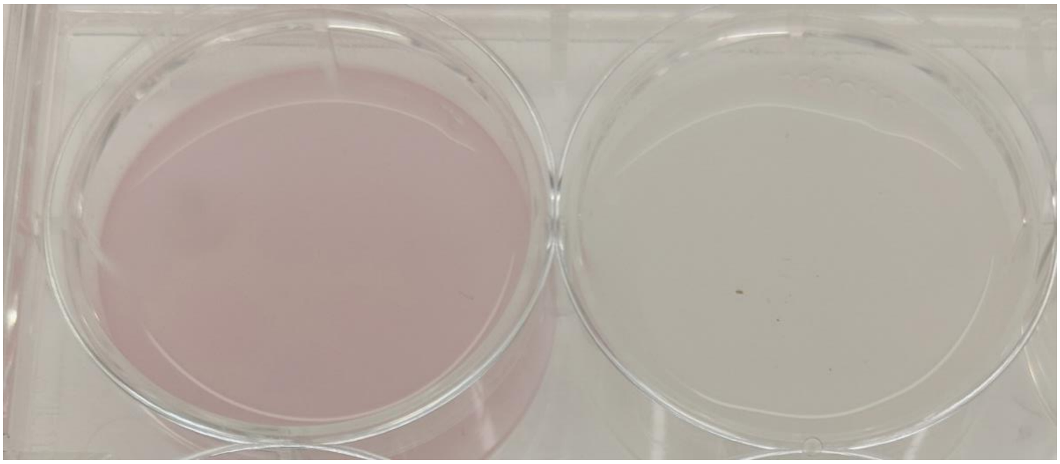

Wild type ∆*pigA*

Supplementary Fig 5.

Prodigiosin production. (A) *S. marcescens* K904 wild type and Prodigiosin mutant (∆*pigA*) flasks were grown overnight in LB. The red color atop the ∆*pigA* mutant is a reflection from the wild type flask. **(B)** Overnight cultures were washed in HEPES buffer and placed in a 12 well plate. The presence of prodigiosin is seen by the red pigmentation of the wild type culture, which is absent in the ∆*pigA* mutant.

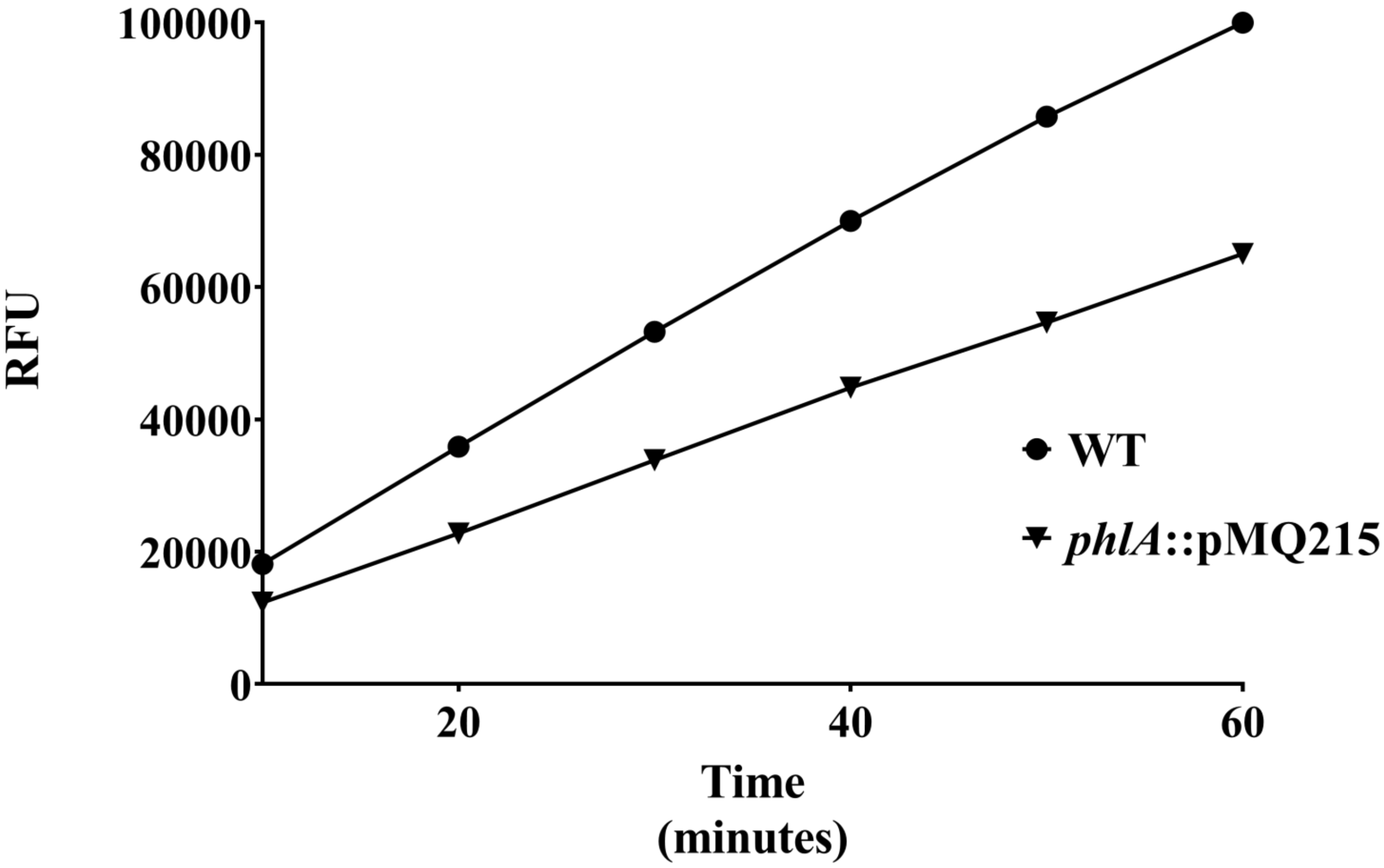

Supplementary Fig 6.
Phospholipase activity. *S. marcescens* K904 (WT) and Phospholipase-A mutant (*phlA*::pMQ215) were grown overnight in LB washed in HEPES buffer (*n* = 1). EnzChek Phospholipase A1 Assay Kit (E10219) from ThermoFisher Scientific was used to measure phospholipase A1 activity. Fluorescence activity was measured using Synergy H1 plate reader from BioTek. Activity was measured every 10 minutes for 1 hour.
